# A Systematic Review: Deep Learning for Analyzing Genomic Data to Discover Evolutionary Patterns

**DOI:** 10.1155/sci5/4286814

**Published:** 2026-07-23

**Authors:** Raha Hassanpour Faramoushjani, Sanam Ansari

**Affiliations:** ^1^ Department of Biology, Yazd University, Yazd, Iran, yazd.ac.ir; ^2^ Department of Software Engineering, T.C İstanbul Beykent University Faculty of Engineering Architecture, Istanbul 34396, Turkey

**Keywords:** deep learning, evolutionary patterns, genomics data, neural networks

## Abstract

Deep learning has been increasingly applied to evolutionary genomics as genomic datasets have grown in scale and complexity. However, the literature encompasses heterogeneous biological objectives, modeling assumptions, and evaluation standards, often treated as a unified field despite important conceptual differences. This study presents a systematic review of research published between 2016 and 2025 on the use of deep learning to identify evolutionary patterns in genomic data. Following a structured screening process, 50 studies were selected for qualitative synthesis. The reviewed applications can be organized into three partially overlapping but conceptually distinct domains: (i) population genetic inference, (ii) phylogenetic reconstruction, and (iii) sequence representation learning using DNA and protein language models. In population genetics, deep learning is predominantly employed within simulation‐based inference frameworks. In phylogenetics, neural architectures are used to approximate or accelerate tree and model inference under defined conditions. In representation learning, models focus on extracting transferable sequence features for downstream evolutionary or functional analyses. Across domains, deep learning provides flexible modeling of complex genomic inputs. Nevertheless, recurring limitations include challenges in interpretability, sensitivity to training assumptions—particularly under simulation‐based settings—heterogeneous evaluation protocols, and substantial computational demands. By organizing the literature using a domain‐aligned framework, this review clarifies domain‐specific strengths, limitations, and research gaps, providing a structured basis for future methodological development in evolutionary genomics.

## 1. Introduction

As a key resource in biology and medicine, genomic data enable deeper understanding of biological processes, including the evolution of species. Accurate analysis of these data supports the identification of evolutionary patterns—such as genetic change through time, mutation and adaptation, and phylogenetic relationships—which in turn informs genetics, personalized medicine, biotechnology, and biological resource management [1, 2].

The rapid adoption of next‐generation sequencing (NGS) has made large volumes of genomic data widely available, accelerating discovery while also creating practical challenges for analysis and interpretation [3]. High dimensionality, heterogeneous evolutionary regimes, and measurement noise can complicate inference. Classical statistical pipelines and traditional machine learning approaches remain essential, but they can face limitations in certain high‐dimensional or highly heterogeneous settings, particularly when strong model assumptions are violated or when flexible representation learning is required [4].

Deep learning has expanded the computational toolkit for analyzing complex, high‐volume data and has been increasingly applied in computational biology and genomics [5]. In evolutionary genomics, deep learning methods have been explored for tasks including selection detection, demographic inference, admixture and hybridization characterization, and phylogenetic reconstruction [6–9]. Importantly, evidence reported across studies is often context‐dependent: performance and computational feasibility depend on the chosen input representations, training regimes, and evaluation designs [10–13].

Despite progress, accurate inference of evolutionary patterns from genomic data remains challenging due to model misspecification risk, computational demands, and evolutionary heterogeneity [8, 14]. Many population‐genetic applications rely on simulation‐based supervised learning, where external validity depends on how well simulated training regimes approximate empirical complexity; distribution shift and demographic or recombination misspecification can therefore degrade generalization [6, 15, 16]. In addition, ongoing debates concern the trade‐off between predictive accuracy and interpretability and the robustness of models when training data or generative assumptions are uncertain [6, 17].

Motivated by these challenges, this review adopts a domain‐aligned perspective that separates biologically distinct objectives and evaluation practices. Applications of deep learning in evolutionary genomics can be grouped into three partially overlapping but conceptually distinct domains: (i) population genetic inference, (ii) phylogenetic reconstruction, and (iii) sequence representation learning through self‐supervised DNA and protein language models. While these domains share computational foundations, they address different biological questions and rely on different modeling and validation frameworks; in the reviewed literature, population genetic inference dominates empirical applications, whereas language models represent a complementary and emerging paradigm [6, 7, 18, 19].

The aim of this systematic review is to critically evaluate deep learning methods applied to discovering evolutionary patterns in genomic data, with emphasis on methodological choices, validation practices, strengths, limitations, and actionable research gaps [6, 9, 11, 20]. By synthesizing evidence across diverse studies and tasks, the review seeks to align computational advances with domain‐specific biological questions and to support more principled method selection and development [6, 7, 12, 16].

This review contributes to the literature in the following ways:

1. Domain‐specific structuring: it organizes the literature into population genetic inference, phylogenetic reconstruction, and language/representation models to reduce cross‐domain conflation and clarify differences in objectives, data representations, and evaluation criteria.
2. Comparative methodological synthesis: it summarizes typical inputs, training paradigms (including simulation‐based training where applicable), and evaluation practices in each domain, highlighting how these choices shape reported results [6–8, 14].
3. Balanced assessment of strengths and limitations: it consolidates recurring limitations—such as distribution shift, computational demands, and interpretability constraints—without overstating generalizability, and it identifies where evidence is most sensitive to modeling assumptions [6, 15, 17].
4. Benchmark‐aware outlook: it highlights the need for transparent reporting and more comparable evaluation designs to improve reproducibility and cross‐study interpretability, and it motivates future directions for robust and biologically credible deployment [9, 18, 20].

This systematic review follows a structured selection process guided by PRISMA and analyzes 50 studies published between 2016 and 2025. The remainder of the study is organized as follows: Section 2 describes the methodology; Section 3 reports domain‐specific results; Section 4 provides a critical synthesis; Section 5 discusses implications, limitations, and future directions; and Section 6 concludes the review.

## 2. Methodology

This study employed a structured systematic review approach to identify and synthesize research on deep learning applications in evolutionary genomics. The review followed established reporting standards to ensure transparency and reproducibility in study selection.

### 2.1. Research Question

The primary research question guiding this review was

How have deep learning methods been applied to identify evolutionary patterns in genomic data, and what are their methodological strengths, limitations, and emerging directions?

This question was operationalized through focused search terms targeting deep learning architectures, genomic data analysis, and evolutionary inference tasks.

### 2.2. Search Strategy and Databases

A systematic literature search was conducted across PubMed/MEDLINE, Web of Science Core Collection, Scopus, IEEE Xplore, bioRxiv, arXiv, and Google Scholar to identify relevant studies published between 2016 and 2025. Boolean operators (AND, OR) and subject filters were applied to retrieve studies focusing on deep learning applications in evolutionary genomics.

Studies were considered eligible if they applied deep learning architectures (e.g., convolutional, recurrent, residual, generative, or hybrid neural networks) to evolutionary genomics tasks, including phylogenetic reconstruction, natural selection detection, admixture inference, mutation rate prediction, or evolutionary‐oriented functional genomic analysis. Studies relying exclusively on traditional machine learning methods, lacking empirical validation, or not directly addressing evolutionary genomics were excluded.

**FIGURE 1:**
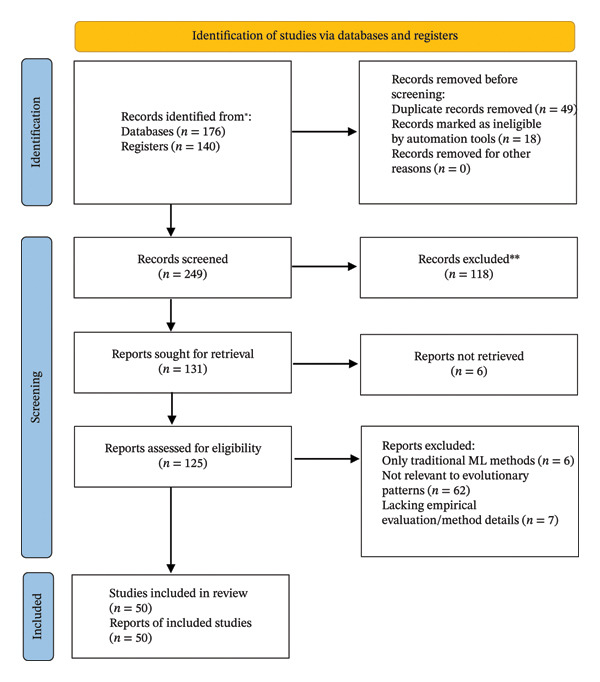
Systematic literature review with PRISMA format.

A total of 176 records were identified through database searches, with an additional 140 studies retrieved via citation chaining. After removing 49 duplicates and 18 ineligible records identified by automated screening, 249 articles remained for title and abstract screening. Of these, 118 were excluded for not meeting the inclusion criteria, leaving 131 studies for full‐text assessment. Six articles could not be retrieved, and 75 were excluded based on relevance and methodological quality. Ultimately, 50 studies were included in the final qualitative synthesis. The detailed selection process is illustrated in Figure 1.

### 2.3. Data Extraction and Coding

For each included study, the following information was systematically extracted:

• Model architecture
• Genomic data representation
• Target evolutionary task
• Evaluation metrics
• Computational characteristics (training and inference considerations)
• Interpretability strategies
• Reported robustness to evolutionary complexity

The analysis was conducted qualitatively and thematically, focusing on methodological trends, comparative strengths, and limitations across studies rather than quantitative meta‐analysis.

Risk‐of‐bias assessment results are provided in Appendix A.

## 3. Result

The results of this systematic review synthesize findings from the selected studies and highlight the main methodological and empirical trends in applying deep learning to evolutionary genomics. Overall, the analysis demonstrates significant progress in model accuracy, scalability, and robustness, while also revealing persistent challenges such as interpretability and generalizability. In the following subsections, the results are presented in detail, beginning with a descriptive overview of the included studies and continuing with a critical synthesis and thematic analysis.

### 3.1. Descriptive Analysis

This section outlines the research landscape of the literature on the use of deep learning to discover evolutionary patterns in genomic data and includes a diverse collection of studies that apply different deep learning architectures to evolutionary genomics problems. The reviewed works cover applications such as phylogenetic inference, natural selection detection, logging, mutation rate prediction, and identification of functional genomic elements using convolutional, recurrent, residual, and generative neural networks. The comparative analysis highlights methodological trends, including the integration of simulation‐based training, diverse data representations, and efforts to increase interpretability and robustness. This combination is crucial for addressing research questions about model capabilities, interpretability, and the management of evolutionary complexity, thereby guiding future methodological developments.

The included studies can be broadly categorized into three primary domains within evolutionary genomics. The first domain, population genetics, comprises studies focused on detecting natural selection, inferring demographic history, admixture, introgression, and polygenic adaptation using deep learning approaches. The second domain, phylogenetic, includes works aimed at inferring evolutionary relationships, reconstructing tree topologies, estimating substitution patterns, and modeling diversification processes. The third domain, referred to here as language and representation learning models, encompasses studies that apply sequence‐based deep learning architectures—often including self‐supervised or large‐scale models—to learn representations from DNA or protein sequences for tasks such as motif discovery, functional element identification, conservation analysis, and sequence‐to‐function prediction. While these domains share methodological foundations, they address distinct biological questions and rely on different evaluation frameworks. Table 1 shows the descriptive analysis of studies.

**TABLE 1:** Descriptive summary of studies.

Study	Model accuracy	Computational efficiency	Data representation	Interpretability	Robustness to evolutionary complexity	Primary domain
Korfmann et al. [6]	High accuracy in detecting selection and demography	Moderate training time; uses simulators for training data	Population genetic data encoded as haplotypes and temporal data	Moderate interpretability; some efforts on network explainability	Handles balancing selection and demographic scenarios well	Population genetics
Yang et al. [21]	High accuracy in tracing genealogy origins across diverse geographic populations	Relatively efficient; deep learning framework optimized for large‐scale genomic datasets.	Genomic variation encoded using sequence‐based and feature extraction approaches	Moderate interpretability; results linked to biologically meaningful evolutionary relationships	Strong robustness; performs well under complex evolutionary patterns such as admixture and mutation	Population genetics
Zhang et al. [10]	Effective in inferring introgression and hybridization	Efficient inference on whole genomes	Sequence alignments from population genomics and assemblies	Limited interpretability; focuses on prediction accuracy	Robust to admixture and hybridization events	Population genetics
Blischak et al. [12]	Accurate hybrid speciation inference using CNNs	Computationally efficient with linked SNPs as images	SNPs encoded as binary images of pairwise divergence	Moderate interpretability via model selection among scenarios	Handles linked data and admixture effectively	Population genetics
Zou et al. [8]	Outperforms traditional methods on heterogeneous data	GPU‐accelerated, faster than ML and BI methods	One‐hot encoded amino acid sequences	Good interpretability; residual networks capture complex signals	Robust to substitution heterogeneity and LBA trees	Phylogenetic
Suvorov et al. [7]	High accuracy in quartet topology inference	Fast inference; robust to gapped and ungapped data	Multiple sequence alignments	Confidence scores more reliable than bootstrap	Robust to bias‐inducing parameter zones	Phylogenetic
Silvestro et al. [14]	Matches or exceeds likelihood‐based inference	Highly scalable to large genomic datasets	Multiple sequence alignments directly analyzed	Provides biologically interpretable patterns	Handles complex rate variation and codon models	Phylogenetic
Lambert et al. [22]	Comparable accuracy to likelihood methods	Faster by orders of magnitude	Phylogenies with trait data	Moderate interpretability; regression‐based	Effective for state‐dependent diversification models	Phylogenetic
Burgstaller‐Muehlbacher et al. [23]	Comparable to likelihood‐based model selection	Significantly faster than traditional methods	Multiple sequence alignments	Limited interpretability; model selection focus	Robust across diverse evolutionary models	Phylogenetic
Chrysostomou [24]	High classification accuracy for host species	Efficient without sequence alignment	Protein sequences (neuraminidase)	Moderate interpretability via classification	Handles viral recombination and evolution	Language/representation model
Guo et al. [25]	Accurate prediction of subgenome dominance in ancient polyploids	Moderate computational demand	DNA sequences and methylation data	Provides mechanistic insights into dominance	Limited for neo/synthetic polyploid genomes	Language/representation model
Fang et al. [26]	Superior mutation rate prediction performance	Efficient with transfer learning	Genomic sequences only	Moderate interpretability; mutation rate profiles	Robust across multiple species and mutation contexts	Language/representation model
Tayara & Chong [27]	Improved functional quantification of noncoding DNA	Moderate computational cost	Raw sequences plus evolutionary information	Enhanced interpretability via motif capture	Handles complex regulatory sequence patterns	Language/representation model
Georgakilas et al. [28]	Outperforms stateof‐the‐art in small RNA loci prediction	Efficient scanning of large genomic regions	Sequence, conservation, and secondary structure	High interpretability; iterative background selection	Robust across RNA classes and species	Language/representation model
Qin et al. [9]	Successful identification of loci under selection	Moderate computational requirements	Genotype data predicting traits and location	Moderate interpretability; locus contribution analysis	Robust to complex spatial selection patterns	Population genetics
Tripathi et al. [11]	Outperforms existing methods in polygenic adaptation detection	Highly efficient with sliding window approach	Genome‐wide SNP data	Moderate interpretability; feature selection included	Robust to demographic history and selection gradients	Population genetics
Xue et al. [13]	High resolution in detecting partial selective sweeps	Efficient with CNN and coalescent simulations	Population genomic data	Limited interpretability; classification focused	Robust to demographic scenarios and sweep types	Population genetics
Hejase et al. [15]	Accurate selection coefficient inference	Moderate computational demand with LSTM	Ancestral recombination graph data	Moderate interpretability; allele frequency trajectories	Handles partial soft sweeps and complex selection	Population genetics
Nguembang et al. [16]	∼90% accuracy in selection classification	Moderate training on simulated data	Genomic windows from population samples	Limited interpretability; classification accuracy emphasized	Robust to neutral and selection processes	Population genetics
Li et al. [29]	Effective in discriminating conserved sequences	Moderate computational cost	Short DNA segments one‐hot encoded	High interpretability; motif visualization	Captures functional conservation patterns	Language/representation model
Shim [30]	Captures allele interactions in viral evolution	Moderate computational demand	Time‐sampled whole‐genome allele frequency data	Moderate interpretability via vector clustering	Accounts for recombination and allele interactions	Population genetics
Sanchez et al. [20]	Flexible for demographic inference tasks	User‐friendly with moderate efficiency	SNP data with customizable preprocessing	Moderate interpretability; task‐agnostic	Robust across demographic scenarios	Population genetics
Yelmen & Jay [18]	Enables data generation and dimensionality reduction	Computationally intensive for generative tasks	Genomic data modeled in latent space	Moderate interpretability; latent space analysis	Handles complex genomic data structures	Cross domain
Wang et al. [31]	High performance in plant genome sequence analysis	Moderate to high computational cost	Multiomics data including sequence and epigenetics	Moderate interpretability; motif mining and design	Robust to diverse plant genomic features	Language/representation model
Bao et al. [32]	Comprehensive coverage of deep learning models	Varies by model; generally efficient	DNA, RNA, and protein data	Moderate interpretability; model‐specific	Addresses livestock genomics challenges	Cross domain
Sun et al. [33]	Benchmarking reveals model strengths and limits	Efficient with optimized training strategies	ATAC‐seq and single‐cell data	High interpretability; sequence‐function models	Specialized for single‐cell and enhancer logic	Language/representation model
Hilten et al. [17]	Quantitative analysis of interpretability designs	Moderate computational demand	Omics data with diverse architectures	High focus on interpretability strategies	Addresses challenges in functional genomics	Language/representation model
Yue et al. [34]	Overview of model strengths for genomic tasks	Varies; emphasizes model‐task fit	Diverse genomic data types	Moderate interpretability; practical considerations	Discusses challenges and future directions	Language/representation model
Routhier and Mozziconacci [19]	Reviews deep learning for genome annotation	Moderate computational requirements	Genomic sequences	Moderate interpretability; functional annotation	Focus on sequence determinants and synthetic design	Language/representation model
Chandrashekar et al. [35]	Highlights AI integration in NGS pipelines	Improves computational time and automation	DNA and RNA sequencing data	Limited interpretability focus	Enhances large‐scale genomic data processing	Cross domain
Alharbi and Rashid [36]	Reviews deep learning in human genomics	Varies; generally efficient	Human genomic data	Moderate interpretability; application overview	Covers under‐ and over‐charted genomic areas	Cross domain
Gupta et al. [37]	Evaluates CNN and RNN for DNA classification	Efficient training and prediction	DNA sequences	Moderate interpretability; model comparison	Addresses classification accuracy and scalability	Language/representation model
Nayak et al. [38]	Discusses DL for NGS biomedical research	Varies; supports large datasets	NGS data for disease and drug discovery	Moderate interpretability; tool overview	Supports infectious disease and WGS analysis	Cross domain
Junjun et al. [39]	Reviews DL‐based variant‐calling methods	Efficient variant detection	Sequencing data	Moderate interpretability; variant classification	Addresses small and structural variants	Cross domain
Durge et al. [40]	Compares genomic sequence processing models	Varies; includes CNN, DBN, MLP	Multiple genomic data types	Moderate interpretability; performance metrics	Recommends models for specific genomic tasks	Cross domain
He et al. [41]	Reviews DL in DNA/RNA motif mining	Models improve with data size	Sequence data with motif features	Moderate interpretability; motif discovery	Highlights challenges and future directions	Cross domain
Zhang et al. [42]	Benchmarks DL algorithms for motif finding	Efficient with diverse datasets	ChIP‐Seq, CLIP‐Seq, cancer, single‐cell data	High interpretability; motif accuracy and scalability	Addresses noisy data and class imbalance	Language/representation model
Liu et al. [43]	Reviews DL applications in genomics	Moderate computational demand	DNA, RNA, protein data	Moderate interpretability; workflow focus	Discusses challenges and future perspectives	Cross domain
Montesinos‐López et al. [44]	Reviews DL in genomic selection	Varies; requires large training data	Genomic prediction data	Moderate interpretability; prediction accuracy	Captures nonlinear patterns in breeding data	Cross domain
Meher [45]	Highlights DL in bioinformatics big data	Efficient for large datasets	Omics and sequence data	Moderate interpretability; application overview	Supports genomic sequence and protein analysis	Cross domain
Abd–Alhalem [46]	Surveys DL for DNA sequence classification	Efficient with GPU acceleration	DNA sequences	Moderate interpretability; architecture overview	Discusses limitations and suggestions	Cross domain
Shen et al. [47]	Brief review of DL in genomic studies	Moderate computational cost	Genomic data	Moderate interpretability; challenges noted	Covers emerging DL techniques	Cross domain
Aggarwal [48]	Reviews ML in genomics data analysis	Efficient for large datasets	Genomic data	Moderate interpretability; potential and challenges	Highlights future directions	Cross domain
Angermueller et al. [49]	Early review of DL in computational biology	Moderate computational demand	Regulatory genomics and imaging data	Moderate interpretability; pitfalls discussed	Focus on large data sets and prediction	Cross domain
Min et al. [50]	Reviews DL in bioinformatics	Varies by architecture	Omics and biomedical data	Moderate interpretability; theoretical issues	Suggests future research directions	Cross domain
Washburn et al. [51]	High accuracy in predicting transcript abundance directly from DNA sequences using deep learning	Moderate efficiency; deep learning models require significant training resources, but inference is relatively fast	Raw DNA sequences encoded (e.g., one‐hot encoding) as inputs to neural networks for transcriptome prediction.	Limited interpretability; some attempts to link sequence motifs to regulatory features, but largely remains a “black box”	Performs well across diverse genomic backgrounds, though robustness depends on training data diversity	Language/representation model
Sidi et al. [52]	High accuracy in predicting gene sequences and capturing codon usage patterns with AI models	Moderate efficiency; training deep models on genomic sequence data requires resources, but inference is relatively efficient	Gene sequences encoded into machine‐readable formats (e.g., codon‐level embedding, sequence encoding) for training	Moderate interpretability; provides insights into codon usage bias, but deeper biological meaning still partially opaque	Robust in handling variability in codon usage across organisms, but sensitive to training dataset composition	Language/representation model
Arnab et al. [53]	High accuracy in detecting natural selection by leveraging spectral analysis of genomic summary statistics	Efficient once summary statistics are computed; spectral analysis reduces dimensionality and computational burden	Uses spectral transforms of genomic summary statistics for compact and informative representation	Relatively high interpretability; spectral components provide biologically meaningful signals of selection	Performs well under diverse demographic histories and selection types, though dependent on quality of summary statistics	Population genetics
Voznica et al. [54]	High accuracy in inferring epidemiological dynamics from phylogenetic trees	Moderate efficiency; training deep neural networks on large phylogenies can be resource intensive	Phylogenetic trees encoded into numerical features suitable for deep learning architectures	Moderate interpretability; some effort toward linking deep features to epidemiological meaning, but still partly “black box”	Strong robustness; effective in capturing outbreak dynamics across complex transmission and evolutionary scenarios	Phylogenetic
Eneli et al. [55]	Promising accuracy in evolutionary inference of malaria vectors using generative models	Moderate to low efficiency; training generative models (e.g., GANs, VAEs) on genomic data can be computationally demanding	Genomic sequences encoded for generative modeling, enabling the synthesis of realistic genomic variation patterns	Moderate interpretability; some insights into generated features, but biological meaning can be opaque	Strong potential robustness, as generative frameworks can capture diverse evolutionary processes, though validation is ongoing	Language/representation model

As presented in Table 1, the studies reviewed in this systematic review fall into three primary domains: population genetics, phylogenetic, and language/representation models. The distribution of studies across these domains illustrates the growing breadth of deep learning applications in evolutionary genomics.

1. Population genetics studies dominate the table, reflecting the widespread use of deep learning methods to analyze evolutionary processes such as selection, demographic history, and gene flow. These studies primarily focus on using genetic data to infer population structure and detect selection signals, with many utilizing simulation‐based models.
2. Phylogenetic studies represent the second‐largest category, focusing on the inference of evolutionary relationships through the reconstruction of phylogenetic trees. These studies highlight how deep learning can improve the accuracy of tree construction and substitution model estimation, particularly when dealing with large and complex datasets.
3. Language/representation models appear as a growing area in evolutionary genomics. These studies leverage deep learning models, particularly self‐supervised learning approaches, to learn representations from genomic sequences. While these models do not directly infer evolutionary relationships, they play a critical role in sequence‐to‐function tasks like motif discovery and protein structure prediction.

Overall, Table 1 highlights the diversity of applications in the field, as well as the different biological questions addressed by each domain. The following sections will delve deeper into the strengths, limitations, and trends within each of these domains.

### 3.2. Critical Analysis and Synthesis

While deep learning approaches in evolutionary genomics share computational foundations, the reviewed studies address biologically distinct objectives. To avoid cross‐domain conflation, Table 2 synthesizes domain‐specific objectives, input representations, training paradigms, evaluation criteria, strengths, and limitations across four categories: population genetics, phylogenetic reconstruction, language/representation models, and cross‐domain studies. The analysis below expands directly on these structured dimensions.

**TABLE 2:** Critical analysis of reviewed papers.

Domain	Primary objective and typical tasks	Typical inputs/representations	Typical training paradigm	Evaluation focus (common in studies)	Key strengths (domain‐specific)	Key limitations/challenges (domain‐specific)
Population genetics	Selection detection; demographic inference; admixture/introgression; polygenic adaptation; population structure and history	SNP windows; haplotypes; allele‐frequency trajectories; divergence matrices; ARG‐derived summaries; population‐genetic feature encodings	Predominantly simulation‐based training (coalescent or forward simulators) + supervised classification/regression; occasional empirical calibration	Accuracy for scenario classification/parameter inference; robustness across simulated scenarios; inference‐time efficiency	Flexible mapping of complex signals; supports diverse encodings; inference often fast once trained	Simulation‐to‐real domain shift; sensitivity to simulator parameters; limited interpretability (often post hoc); high training cost due to simulations and tuning
Phylogenetic	Tree topology inference; substitution‐pattern inference; diversification/state‐dependent models; tree‐based epidemiological dynamics	Multiple sequence alignments (MSAs); alignment‐derived tensors; tree encodings; trait + tree features	Supervised learning on simulated or benchmarked phylogenetic data; neural networks as approximators of classical inference	Topology accuracy; robustness to substitution heterogeneity; inference speed comparison; confidence reliability	Potential acceleration after training; robustness in heterogeneity regimes	Scalability constraints for large trees; sensitivity to alignment properties and taxon sampling; heterogeneous benchmarking; limited interpretability
Language/representation models	Learning sequence representations; motif discovery; functional element identification; conservation‐related analysis; sequence‐tofunction prediction; multiomics modeling	Raw DNA/protein sequences; embeddings; conservation/structure features; methylation/epigenetic inputs; single‐cell assay data	Self‐supervised or transfer learning + downstream supervised tasks; large‐scale pretraining where available	Task‐specific predictive metrics; transferability; motif/feature visualization	Strong feature learning; reusable embeddings; useful for sequence‐tofunction pipelines	Not inherently evolutionary inference unless integrated; metrics not directly comparable across domains; interpretability challenges for large models; high training cost
Cross‐domain	Reviews/benchmarks across genomics tasks; integrative methods; generative models for dimensionality reduction or data synthesis	Diverse inputs (sequence, omics layers, summary features); latent representations; mixed‐input pipelines	Heterogeneous: surveys, benchmarks, generative modeling, integrative frameworks	Structured comparison; breadth of coverage; interpretability emphasis	Maps the methodological landscape; highlights transferable ideas	Risk of over‐generalization; heterogeneous evaluation; generative validity and biological plausibility challenges

Table 2 restructures the critical analysis by the three primary domains used in Table 1 (primary domain), separating population genetic inference, phylogenetic reconstruction, and language/representation learning models to avoid cross‐domain conflation and to clarify domain‐specific strengths, limitations, and evaluation practices.

#### 3.2.1. Population Genetic Inference

As summarized in Table 2, studies categorized under population genetics primarily focus on inference tasks such as selection detection, demographic history reconstruction, admixture and introgression detection, polygenic adaptation, and population structure analysis.

These studies commonly operate on SNP windows, haplotypes, allele‐frequency trajectories, divergence matrices, ancestral recombination graph (ARG)–derived summaries, or other population‐genetic encodings. Such representations aim to capture structured signals produced by evolutionary processes at the population level.

A defining methodological feature in this domain is simulation‐based training. Most models are trained using coalescent or forward‐time simulators to generate labeled evolutionary scenarios, followed by supervised classification or regression. In some cases, limited empirical calibration is incorporated.

Evaluation typically emphasizes accuracy in scenario classification or parameter estimation, robustness across simulated evolutionary regimes, and—in some studies—inference‐time efficiency once models are trained.

Deep learning offers flexibility in mapping heterogeneous population‐genetic representations to inferred evolutionary outputs. Once trained, models often provide rapid inference relative to repeated likelihood‐based procedures.

The central challenge is generalizability. Model performance depends strongly on how well simulated training regimes approximate real evolutionary complexity. Domain shift between simulated and empirical data can reduce external validity. Interpretability is frequently limited to post hoc feature attribution. Moreover, computational cost in this domain is often dominated by simulation generation and hyperparameter tuning, making it important to distinguish between training cost and inference cost.

Critical evaluation in this domain should prioritize transparency in simulator parameterization, assessment of robustness under mis‐specified scenarios, and explicit reporting of training versus inference computational demands.

#### 3.2.2. Phylogenetic Reconstruction

According to Table 2, the phylogenetic domain includes studies addressing tree topology inference, substitution‐pattern modeling, diversification processes, and tree‐based epidemiological dynamics.

Inputs commonly include multiple sequence alignments (MSAs), alignment‐derived tensors, encoded phylogenetic trees, and trait‐plus‐tree features.

Many approaches rely on supervised learning using simulated or benchmarked phylogenetic datasets. Neural networks frequently function as approximators or accelerators of classical likelihood‐based inference steps.

Evaluation commonly centers on topology accuracy, robustness to substitution heterogeneity or bias‐prone regimes, inference speed comparisons, and the reliability of model‐derived confidence scores.

Deep learning models can accelerate inference once trained and may demonstrate robustness in regimes that are challenging for some classical assumptions, such as heterogeneous substitution processes.

Limitations are domain‐specific. Some architectures are constrained by fixed input structures (e.g., restricted taxon counts), and scalability to large phylogenies can be limited. Performance may be sensitive to alignment properties and taxon sampling. Additionally, benchmarking protocols vary across studies, complicating direct comparison of reported results.

Meaningful evaluation in this domain requires standardized benchmarking conditions, explicit separation of training and inference costs, and careful interpretation of confidence metrics relative to classical phylogenetic uncertainty measures.

#### 3.2.3. Language and Representation Learning Models

The third domain in Table 2 includes language and representation models, which focus on learning sequence‐level embedding rather than directly estimating evolutionary parameters or reconstructing trees.

Typical tasks include motif discovery, functional element identification, conservation‐related analysis, sequence‐to‐function prediction, and, in some cases, multiomics sequence modeling.

Models operate on raw DNA or protein sequences, often encoded via one‐hot representations or embeddings, and may incorporate conservation, structural, epigenetic, or single‐cell assay features.

Many approaches employ self‐supervised or transfer learning strategies, followed by downstream supervised tasks. Large‐scale pretraining is used where data availability permits.

Evaluation typically relies on task‐specific predictive metrics (e.g., classification or regression accuracy), transferability across datasets or tasks, and interpretability analyses such as motif visualization.

This domain benefits from strong feature learning from large sequence corpora and the development of reusable embeddings that support diverse downstream analyses.

These models do not inherently constitute evolutionary inference frameworks unless explicitly integrated with evolutionary modeling assumptions. Their evaluation metrics are not directly comparable with those used in population genetics or phylogenetics. Interpretability can be challenging for large models, and pretraining can incur substantial computational costs.

Representation models should be interpreted as complementary tools that enhance sequence‐level understanding and downstream analysis, rather than direct substitutes for population‐genetic or phylogenetic inference.

#### 3.2.4. Cross‐Domain Studies and Integration Opportunities

Table 2 also identifies cross‐domain studies, including reviews, benchmarks, integrative pipelines, and generative modeling frameworks that span multiple genomic subfields.

These works often aim to provide structured comparisons, develop generalized pipelines, or propose methods intended to operate across heterogeneous genomic inputs.

Methodologies vary widely, including benchmarking studies, integrative modeling approaches, and generative data augmentation frameworks. Evaluation typically emphasizes breadth of coverage and methodological comparison.

Cross‐domain studies help map the methodological landscape and highlight transferable ideas, such as interpretability frameworks, representation learning strategies, and generative augmentation techniques.

A key risk is over‐generalization: Heterogeneous evaluation settings and domain‐specific objectives make “one‐size‐fits‐all” conclusions unreliable. Generative approaches additionally require careful validation to ensure biological plausibility.

While domain separation is necessary for analytical clarity, methodological transfer between domains—such as combining pretrained representations with simulation‐based frameworks—represents a promising direction for future integration without conflating biological objectives.

### 3.3. Thematic Review of the Literature

While Section 3.2 analyzed the literature according to biological domains, this section reorganizes the same body of work thematically across domains. Therefore, themes discussed here may span more than one domain but are interpreted in light of the distinctions clarified above.

Given the interdisciplinary nature of applying deep learning to genomic data analysis to discover evolutionary patterns, previous studies have approached this issue from a variety of approaches. The thematic review allows us to structure the existing literature, identify the main areas of deep learning use, and highlight conceptual trends emerging from previous research. The literature on the applications of deep learning to discover evolutionary patterns in genomic data reveals several dominant themes. The main focus is on the development and evaluation of diverse deep learning architectures—such as convolutional, recurrent, and residual neural networks—designed to infer evolutionary relationships and detect selection signals from genomic sequences. Another key theme involves the integration of simulation‐based training with empirical genomic data to enhance model robustness and interpretability. In addition, the investigation of genomic data representation and preprocessing techniques to improve predictive accuracy appears to be crucial. Challenges related to model interpretability, scalability, and handling of complex evolutionary scenarios such as hybridization and polyploidy are also often discussed, along with the evolving role of generative models and large‐scale data integration.

Table 3 provides a thematic review of selected studies, showing that research in this area can be grouped into several main categories. As can be seen, the majority of studies have focused on the development and application of deep learning architectures for evolutionary inference (e.g., phylogenetic tree construction, selection detection, and genomic sequence classification). This suggests that researchers are primarily looking to replace traditional methods with more accurate and scalable architectures.

**TABLE 3:** Thematic review of studies.

Theme	Appears in	Theme description
Deep learning architectures for evolutionary inference	28/50 papers	Several studies have developed and tested deep learning models, including CNNs, RNNs, residual networks, and hybrids, for inferring phylogenetic trees, detecting selection, and classifying genomic sequences. These architectures have shown improved accuracy and robustness over traditional approaches, especially in handling nonlinear evolutionary processes and heterogeneous substitution patterns [6–8, 12, 41]. Residual neural networks show particular promise in quadruple tree inference and handling substitution heterogeneity [8]
Simulation‐based training and empirical data integration	25/50 papers	The use of convolutional and forward simulations to generate training datasets under complex evolutionary scenarios is widespread and facilitates model training for population inference, selection detection, and hybridization analysis. Integration with real genomic data increases the applicability of the model and allows the detection of signals such as recent balancing selection, inbreeding, and multigene adaptation [6, 9, 10, 13, 15]. Such approaches help to overcome the limitations of parametric models and improve generalizability
Genomic data representation and preprocessing strategies	21/50 papers	Effective encoding of genomic data, including one‐hot encoding of sequences, matrix representation of SNP data, and inclusion of evolutionary conservation and secondary structure, is a recurring theme. Preprocessing methods significantly affect model performance in predicting regulatory elements, mutation rates, and gene expression from genomic sequences [6, 27–29, 41]. New pipelines automate feature extraction and background selection to optimize training [28]
Interpretability and robustness of deep learning models	19/50 papers	Research emphasizes the need to clarify how models extract evolutionary signals and ensure robustness to uncertain or noisy training data. Techniques such as network kernel inspection, model weight inspection, and feature importance analysis are used to improve biological interpretability. Challenges include overcoming model opacity and avoiding overfitting, especially in complex evolutionary contexts [6, 8, 17, 41, 42]
Handling complex evolutionary phenomena	15/50 papers	Studies explore the applications of deep learning in complex patterns such as hybridization, introgression, polyploidy, and spatially variable selection. CNN‐based methods have been developed to infer hybrid speciation, while models have been applied to infer subgenome dominance and detect multigene adaptation across populations. This highlights the ability of deep learning to solve lattice and nonlinear evolutionary processes [9–12, 25]
Generative models and dimensionality reduction in genomics	10/50 papers	Deep generative models, including autoencoders and generative adversarial networks (GANs), are emerging as powerful tools for dimensionality reduction, data synthesis, and feature learning in evolutionary genomics. These models help simulate realistic genomic data and extract latent representations that capture evolutionary structure, complementing discriminative approaches [6, 18, 28]
Large‐scale, multiomics and cross‐species applications	9/50 papers	The application of deep learning extends to the integration of multiomics and cross‐species analyses, such as gene expression landscape prediction across species and cross‐species identification of small RNAs. These approaches enhance the understanding of conserved regulatory elements and evolutionary patterns beyond single‐species contexts [28, 31]
Scalability and computational efficiency	8/50 papers	Given the computational requirements, several studies emphasize the increased efficiency of GPU‐accelerated deep learning and automated pipelines. Models such as RAISING demonstrate large‐scale genome‐wide implementation with reduced computational time while maintaining high accuracy in detecting adaptive signals [8, 11, 20]
Comparative evaluation of discriminative vs. generative models	7/50 papers	Some works compare discriminative deep learning methods with generative approaches and evaluate their relative strengths in evolutionary inference tasks. While discriminative models excel in classification and prediction, generative models play a role in flexible simulation and hidden feature extraction, suggesting complementary roles [6, 18, 34]
Challenges and future directions in deep learning for evolutionary genomics	6/50 papers	Reviews and perspectives underscore challenges such as model interpretability, data imbalance, hyperparameter tuning, and the need for more biologically informed architectures. They advocate for integrating evolutionary knowledge, improving generalizability, and expanding applications to diverse genomic contexts and data types [17, 36, 43, 47]

On the other hand, the use of simulated data alongside experimental data is seen as a major recurring theme in studies. This approach allows for the investigation of complex evolutionary scenarios but at the same time presents challenges in terms of generalizability to real data.

Another important topic reviewed in the table is data representation and preprocessing methods, which play a key role in improving model performance. The use of innovative representations such as single‐letter encoding of sequences, SNP matrices, and even structural and conservation information has increased the accuracy of the models.

The section on interpretability and robustness of models also shows that although some efforts have been made to clarify the decisions of networks, this area remains one of the biggest challenges for deep learning in biology.

Other studies have focused on complex evolutionary phenomena (such as hybridization, introgression, and polygenic traits) and have shown that deep learning has the ability to model these nonlinear patterns better than classical statistical methods.

Looking ahead, the areas of generative models and dimensionality reduction as well as multiomics data integration represent emerging opportunities that can provide a deeper understanding of evolutionary processes, although they are still in their early stages of development. Overall, Table 3 shows that although a variety of research paths have been pursued in this area, three main axes—developing architectures, simulating and representing data, and improving interpretability—have played the most important role in advancing deep learning in evolutionary genomics.

**FIGURE 2:**
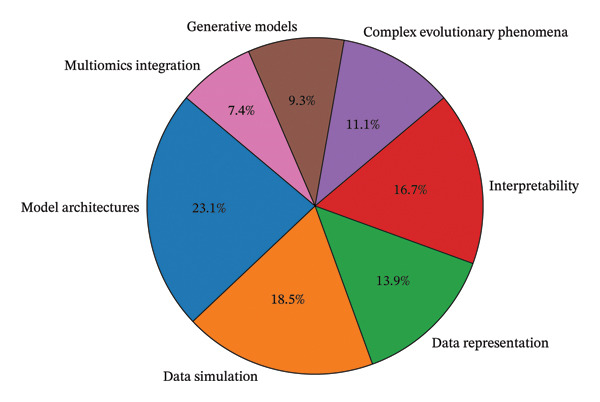
Thematic distribution of reviewed papers.

Figure 2 shows the thematic distribution of the selected studies. As is clear, the most research focus has been on the development of deep learning architectures (23.1%), which indicates the importance of innovation in model design for the analysis of evolutionary genomic data. After that, data simulation (18.5%) and model interpretability (16.7%) have the largest contributions; this shows that, in addition to model accuracy, the ability to represent complex evolutionary scenarios and clarify the results are among the main priorities of researchers.

Topics such as data representation (13.9%) and complex evolutionary phenomena (11.1%) also constitute an important part of the efforts and have helped to increase the accuracy and applicability of models in real conditions. In contrast, emerging topics such as generative models (9.3%) and multiomics data integration (7.4%) have had a smaller share, but given recent trends, they are expected to receive more attention in the coming years. Overall, this distribution shows that research has gradually moved toward newer and more multilayered topics such as multiomics and generative models, in addition to focusing on improving architectures and data simulation.

### 3.4. Literature Review Based on Time

Although the chronological evolution of the literature reflects a continuous expansion of deep learning in evolutionary genomics, the dominance of specific domains has shifted over time. In early years, foundational sequence‐based and representation‐focused studies were more prominent, whereas later periods saw rapid expansion in population genetic inference and phylogenetic reconstruction. More recently, language and generative models have gained visibility, particularly in multiomics and large‐scale genomic applications.

Table 4 shows a description of review papers based on their publication years.

**TABLE 4:** Chronological review of literature.

Year range	Research direction	General description	Description by domain
2016–2017	Foundational deep learning applications in genomics	Initial studies explored the potential of deep learning models such as convolutional neural networks to interpret genomic sequences, identify functional elements, and predict conservation. Emphasis was placed on leveraging sequence motifs and regulatory elements, establishing deep learning as a promising tool for genomics beyond traditional methods. Early frameworks also adapted natural language processing techniques to model viral genome evolution	During this foundational phase, most contributions were aligned with what is now categorized as language/representation models, focusing on sequence interpretation and regulatory genomics rather than explicit evolutionary parameter inference
2018–2019	Deep learning for phylogenetic inference and population genetics	This period marked the application of deep residual networks and CNNs to infer phylogenetic tree topologies with improved accuracy under complex evolutionary models. Deep learning methods were increasingly used for detecting selective sweeps, mutation rate prediction, and elucidating sequence functions, often incorporating simulation‐based training. Tools began to address challenges such as substitution heterogeneity, incomplete lineage sorting, and partial sweeps with notable advances in model robustness and scalability	This period marked a clear rise in population genetic and phylogenetic applications, with simulation‐based inference and topology prediction becoming central research themes
2020–2021	Expanding deep learning architectures and interpretability	Research diversified into integrating recurrent and residual networks for complex evolutionary pattern recognition, including admixture and introgression inference. There was a growing focus on model interpretability, benchmarking of motif‐finding algorithms, and coupling stochastic simulations with deep learning to estimate molecular evolution parameters. Additionally, frameworks for genomic selection and variant calling began to harness deep learning to capture nonlinear relationships in large datasets	Population genetic inference became the dominant domain in terms of application diversity, while phylogenetic deep learning matured in methodological sophistication
2022–2023	Integration of simulation, generative models, and multimodal data	Recent works advanced the coupling of deep learning with stochastic simulations and ancestral recombination graphs to improve selection inference and polygenic adaptation detection. Generative models gained prominence for data augmentation and dimensionality reduction in evolutionary genomics. Cross‐species predictive models and multibranch architectures that integrate sequence, structure, and conservation information emerged, enhancing precision in functional element identification and evolutionary event characterization	The literature diversified substantially during this period, with population genetics still dominant numerically, but language/representation models gaining momentum through self‐supervised and generative approaches
2024–2025	Large‐scale models and automation in genomic evolutionary analyses	The latest research emphasizes automation, scalability, and the application of large language models and deep generative models for interpreting complex genomic and epigenomic data. Advances include improved prediction of subgenome dominance, codon usage patterns, and rapid model selection without explicit likelihood calculations. Benchmarking frameworks for interpretability and sequence‐function prediction have been developed to guide future model design and deployment in evolutionary genomics	In the most recent phase, language/representation models and multiomics integration appear increasingly prominent, reflecting a shift toward large‐scale representation learning and automated genomic analysis frameworks

Table 4 provides a chronological review of the research literature, showing how the use of deep learning in evolutionary genomics has evolved over the past decade. In the early years (2016–2017), the focus was on testing the initial capacity of neural networks (especially CNNs) to identify sequence motifs and conserved elements, and this period was more foundational.

In 2018–2019, the scope of applications expanded, and deep networks were used for phylogenetic inference and population genetic analysis. At this stage, the use of simulated data to train models and detect natural selection or predict mutation rates became a focus.

In 2020–2021, research focused on diversifying architectures (including RNNs and ResNets) as well as increasing the interpretability of models. The use of stochastic simulations combined with deep learning to estimate evolutionary parameters and apply them to new tasks such as selection genomics and variant calling also grew during this period.

From 2022 onwards, the integration of experimental and simulated data with generative and multibranch models expanded. Generative models were used in particular to reduce dimensionality and generate new data. At the same time, attention to multiomics and cross‐species prediction increased, indicating a move toward more multilayered and realistic applications.

Finally, the last years (2024–2025) are characterized by a focus on scalability, automation, and the use of large language models and advanced generative models. This course shows that the field of deep learning in evolutionary genomics has reached a stage of relative maturity, and its future path will be toward broader applications, more efficient models, and more interpretable frameworks.

**FIGURE 3:**
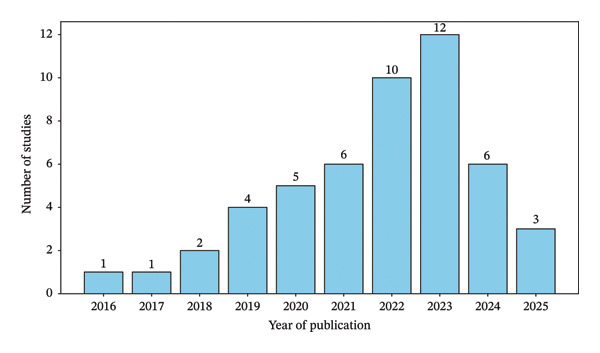
Distribution of publication years.

Figure 3 shows the growth trend in the number of peer‐reviewed articles from 2016 to 2025. As can be seen, in the early years (2016 and 2017), the number of studies was very limited, with only one or two articles published. Since 2018, the trend of research publication has accelerated, and especially since 2019, we have seen a significant increase in the number of articles.

The years 2022 and 2023 are considered the peak of research activity in this field, with the highest number of articles published in this period. This growth can be attributed to the advancement of computational tools, the increased access to large‐scale biological data, and the increasing attention to the applications of deep learning in evolutionary genomics.

In recent years (2024 and 2025), although the number of articles has decreased slightly compared to the peak in 2023, the level of scientific activity remains high. This indicates the consolidation of deep learning’s position in this field and the gradual movement of research from the quantitative growth stage toward qualitative deepening and more practical studies.

**FIGURE 4:**
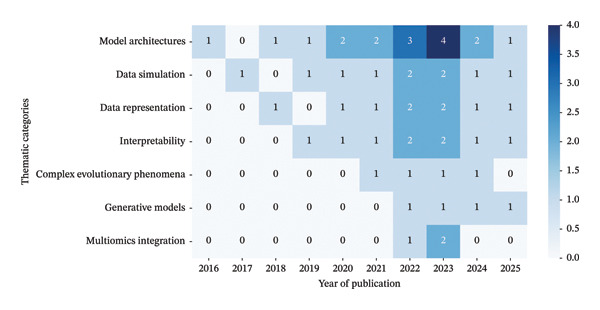
Temporal evolution of research themes across the reviewed studies (heatmap).

Figure 4 shows the trend of the topic distribution of reviewed articles during the years 2016–2025. As can be seen, in the early years (2016–2018) the focus of studies was very limited and mainly focused on model architectures and to some extent data representation. From 2019 onwards, newer areas such as data simulation and interpretability also entered the research literature.

The turning point occurred in the years 2020–2022, when, in addition to an increase in the number of studies, the scope of topics also expanded, and areas such as complex evolutionary phenomena and generative models were also considered. The years 2022 and 2023 show the greatest diversity and volume of studies; in particular, the simultaneous attention to multiomics integration has indicated a movement of research toward multilayered analyses and combined data.

In recent years (2024–2025), although the topic diversity has been maintained, the number of studies has slightly decreased. This likely reflects a shift from a quantitative growth phase to a more qualitative focus on emerging topics such as generative models and multiomics.

Overall, this heatmap illustrates the evolution of research from a narrow focus on architectures in the early years to a broadening of the spectrum of topics in recent years, a trend that reflects the maturity and sophistication of the use of deep learning in evolutionary genomics.

### 3.5. Studies Analysis Based on Agreement and Divergence

Across the reviewed studies, there is broad consensus that deep learning models, particularly convolutional and residual neural networks, show great potential in uncovering complex evolutionary patterns from genomic data and often outperform traditional methods in terms of accuracy and scalability. Most studies agree on the advantages of simulation‐based training data for capturing evolutionary complexity and the need for interpretable models to provide biological insights. However, there is disagreement about the best architectures for specific tasks, the trade‐offs between discriminative and generative approaches, and how to handle heterogeneous evolutionary processes such as induction and selection. These disagreements often stem from differences in study focus, data types, and evolutionary scenarios modeled, as well as different priorities in computational efficiency versus interpretability.

The reviewed literature demonstrates both substantial agreement and notable divergence, but these patterns are domain‐dependent. Therefore, agreement and divergence are examined at two levels: (i) within‐domain consensus and (ii) cross‐domain methodological differences. Table 5 shows the common and different views of research on the application of deep learning in evolutionary genomics.

**TABLE 5:** Agreement and divergence across studies.

Analytical dimension	Population genetics	Phylogenetic	Language/representation	Cross‐domain observation
Core objective	Parameter and scenario inference	Tree topology and substitution modeling	Sequence representation and functional prediction	Objectives are not directly interchangeable
Training paradigm	Mostly simulation‐based supervised learning	Simulated or benchmarked supervised learning	Self‐supervised + downstream supervised tasks	Training philosophies differ substantially
Evaluation metrics	Classification accuracy, parameter error	Topology accuracy, robustness tests	Task‐specific predictive metrics	Metrics not directly comparable
Interpretability	Post hoc feature attribution; locus‐level insights	Confidence estimation; limited mechanistic insight	Motif/feature visualization; embedding analysis	Interpretability definitions differ
Computational profile	Heavy simulation + training; fast inference	Training cost varies; potential inference acceleration	High pretraining cost (large models)	Cost structures are domain‐specific
Major limitation	Domain shift from simulation	Scalability to large trees	Lack of explicit evolutionary grounding	No unified benchmarking framework

Across population genetic studies, there is broad agreement that deep learning models—particularly CNN‐based and hybrid architectures—are effective in detecting selection signatures, demographic patterns, and admixture scenarios under complex evolutionary simulations. Most studies rely on simulation‐based training and acknowledge its necessity for modeling nonlinear evolutionary processes. There is also general consensus that inference time is typically efficient once models are trained. However, many studies recognize the risk of domain shift between simulated and empirical data and the limited interpretability of trained networks.

Within phylogenetic applications, there is agreement that deep learning can approximate or accelerate topology inference and substitution modeling under certain regimes. Studies converge on the importance of robustness to substitution heterogeneity and biased parameter zones. However, scalability to larger phylogenies and standardized benchmarking remain recurring concerns.

Studies in the language modeling domain agree that deep neural architectures are highly effective at learning sequence‐level representations useful for motif discovery, regulatory prediction, and sequence‐to‐function tasks. There is also agreement that transfer learning and large‐scale pretraining enhance generalization across tasks. However, many acknowledge limited direct interpretability and the absence of explicit evolutionary modeling in most architectures.

Despite shared computational foundations, significant divergence exists across domains:

• Objective divergence: Population genetics seeks parameter inference; phylogenetics seeks tree reconstruction; representation models focus on feature learning.
• Evaluation divergence: Metrics are not directly comparable across domains.
• Training paradigm divergence: Simulation‐based supervision dominates population genetics; self‐supervised learning is more common in representation models.
• Interpretability divergence: Biological interpretability has different meanings across domains.
• Computational trade‐offs: Training cost structures differ significantly.

These divergences suggest that deep learning in evolutionary genomics should not be treated as a single unified methodological field, but rather as a set of computationally related yet biologically distinct research programs.

## 4. Theoretical and Practical Implications

The integration of deep learning into evolutionary genomics does not represent a single methodological shift, but rather a set of domain‐specific adaptations across population genetics, phylogenetic reconstruction, and sequence representation learning. As structured in Table 2 and discussed in Section 3, these domains differ in biological objectives, input representations, training paradigms, and evaluation criteria. Consequently, their theoretical and practical implications must be interpreted within domain‐appropriate contexts.

### 4.1. Theoretical Implications

#### 4.1.1. Population Genetic Inference

In population genetics, deep learning is predominantly embedded within simulation‐based inference frameworks. By learning mappings between simulated evolutionary scenarios and inference targets (e.g., selection classes or demographic parameters), neural models provide flexible approximations in settings where explicit likelihoods are difficult to specify or computationally expensive [6, 15].

The theoretical implication is not the replacement of population‐genetic modeling but a shift toward amortized inference strategies: Models learn scenario‐to‐parameter mappings during training and subsequently apply them rapidly. However, this paradigm also shifts epistemic responsibility toward the realism and coverage of simulation regimes. If training simulations inadequately represent evolutionary heterogeneity, the learned mapping may lack external validity.

Thus, the theoretical contribution of deep learning in this domain depends critically on how simulation assumptions are constructed, validated, and stress‐tested.

#### 4.1.2. Phylogenetic Reconstruction

In phylogenetics, deep learning methods are often proposed as accelerators of classical likelihood‐based procedures [8, 14]. Neural networks can learn to predict tree topologies or model characteristics under specific simulated regimes, potentially reducing repeated likelihood evaluations once trained.

The theoretical implication here is more computational than epistemological: Deep learning may function as a surrogate for parts of the inference pipeline. However, uncertainty interpretation remains domain‐specific. Confidence scores produced by neural networks are not automatically equivalent to bootstrap or Bayesian posterior support and must be evaluated accordingly.

Thus, theoretical progress in this domain hinges on clarifying the relationship between neural confidence and phylogenetic uncertainty.

#### 4.1.3. Language and Representation Learning Models

In contrast to direct inference tasks, representation models focus on learning sequence‐level embedding from DNA or protein data [17, 25]. These models often employ self‐supervised or transfer learning strategies and are evaluated on downstream predictive tasks.

The theoretical implication is a shift toward representation‐first genomics, where biologically meaningful structure is captured implicitly within embedding. However, such representations do not inherently encode evolutionary mechanisms unless explicitly integrated with evolutionary modeling assumptions. Therefore, theoretical interpretation requires careful distinction between predictive utility and mechanistic inference.

### 4.2. Practical Implications

The practical value of deep learning in evolutionary genomics depends on how models are trained, evaluated, and reported. These considerations vary substantially across domains.

#### 4.2.1. Distinguishing Training Cost From Inference Cost

Across domains, deep learning models may provide rapid inference once trained. However, training can be computationally demanding—particularly in population genetics, where large simulation corpora must be generated [6, 9], and in representation learning, where large‐scale pretraining may be required [25].

Practical reporting should therefore distinguish the following:

1. Data generation or simulation time
2. Model training time
3. Inference time
4. Compute environment and hardware requirements

Failure to separate these components can produce misleading impressions of scalability or efficiency.

#### 4.2.2. Validation Under Distribution Shift and Evolutionary Complexity

Robustness under distribution shift is a domain‐dependent concern.

In population genetics, simulation‐to‐reality gaps pose a major challenge [6, 15]. Evaluation should include stress testing under demographic misspecification, recombination variation, and selection heterogeneity.

In phylogenetics, benchmarking should span diverse alignment regimes, taxon sampling schemes, and substitution heterogeneity conditions [8, 14].

In representation learning, practical deployment depends on transferability across organisms and tasks, as well as the biological interpretability of learned features [17, 33].

Robust validation frameworks and transparent benchmarking are therefore essential for domain‐appropriate credibility.

#### 4.2.3. Balanced Interpretation and Structured Comparison

Credibility improves when studies avoid qualitative claims of “overall superiority” and instead report domain‐specific objectives, input structures, evaluation metrics, and limitations explicitly.

Because evaluation metrics differ fundamentally across the three domains—parameter estimation accuracy in population genetics, topology accuracy in phylogenetics, and task‐specific prediction metrics in representation learning—cross‐domain performance comparisons can be misleading. Structured domain‐aligned synthesis, such as that provided in Tables 2, 6, and 7, facilitates more precise interpretation.

**TABLE 6:** Domain‐specific limitations of deep learning in evolutionary genomics.

Domain	Limitation category	Description	Representative studies
Population genetics	Simulation‐to‐reality gap	Dependence on simulated training data may reduce generalizability to empirical genomic datasets	[6, 9, 15]
Population genetics	Computational burden	Large simulation corpora and hyperparameter tuning increase training cost	[11, 20]
Phylogenetic	Fixed input constraints	Quartet‐based or fixed‐topology architectures limit scalability to large trees	[7, 8]
Phylogenetic	Benchmark heterogeneity	Variation in alignment properties and evaluation criteria reduces comparability	[14]
Representation/language models	Lack of evolutionary grounding	Optimization focuses on predictive tasks without explicit evolutionary constraints	[25, 33]
Representation/language models	High pretraining cost	Large‐scale models require substantial computational resources	[17, 51]
Cross‐domain	Lack of standardized benchmarks	The absence of unified benchmarks limits reproducibility and fair comparison	[32, 33]

**TABLE 7:** Domain‐specific gaps and future research directions.

Domain	Research gap	Future direction	Representative references
Population genetics	Simulation‐to‐reality gap	Hybrid empirical–simulation training and ARG‐based representations	[6, 9, 15]
Phylogenetic	Scalability to large trees	Permutation‐invariant and graph‐based neural architectures	[8, 14]
Representation/language models	Lack of evolutionary grounding	Evolution‐aware pretraining and mechanistic embedding validation	[17, 25, 33]
Cross‐domain	Absence of standardized benchmarks	Unified benchmarking, statistical testing, transparent reporting	[32, 33]

#### 4.2.4. Adoption in Applied Contexts

Applied areas such as conservation genomics, agriculture, and pathogen surveillance may benefit from deep learning when pipelines are reproducible, computational requirements are transparent, and outputs are interpretable for biological decision‐making [9, 20].

In practice, the most sustainable adoption scenarios appear to be those where deep learning complements existing evolutionary modeling workflows—accelerating repeated analyses, enabling screening across large datasets, or providing enriched sequence representations—while final conclusions remain anchored in domain‐appropriate validation and uncertainty assessment.

## 5. Limitation, Gaps, and Future Research Directions

This section outlines key limitations of the current deep learning approaches in evolutionary genomics and suggests promising directions for future research. Based on the findings in Section 3 and the insights from Tables 6 and 7, we also discuss the methodological gaps that hinder the widespread application of these methods.

### 5.1. Limitations

Despite the substantial methodological advances reviewed in the preceding sections, the limitations identified in the literature are not uniform across domains. Rather, they reflect fundamental differences in biological objectives, input representations, training paradigms, and evaluation strategies across population genetic inference, phylogenetic reconstruction, and representation learning frameworks. Accordingly, the following analysis restructures the identified limitations in a domain‐specific manner to clarify methodological boundaries, avoid cross‐domain conflation, and provide a more balanced and analytically grounded assessment of current deep learning applications in evolutionary genomics.

#### 5.1.1. Population Genetic Inference

In population genetic applications, deep learning models are predominantly embedded within simulation‐based inference pipelines. A primary limitation is the simulation‐to‐reality gap, where models trained on coalescent or forward simulations may fail to generalize to empirical genomic data if demographic heterogeneity, recombination structure, or selection regimes are insufficiently represented [6, 9, 15]. Model generalizability therefore depends critically on the realism and coverage of simulation regimes. In addition, parameter misspecification and limited robustness to demographic shifts may lead to biased inference. While inference time is often efficient once trained, the generation of large simulated training corpora and hyperparameter optimization impose substantial computational cost [6, 11, 20]. Interpretability also remains limited, as many architectures provide post hoc feature attribution rather than mechanistic evolutionary explanations [17].

#### 5.1.2. Phylogenetic Reconstruction

Within phylogenetic reconstruction, deep learning methods are often designed to approximate or accelerate classical likelihood‐based inference. However, several domain‐specific constraints persist. Some neural architectures rely on fixed input structures (e.g., quartet‐based models), limiting scalability to larger taxon sets [7, 8]. Scalability to large phylogenies and heterogeneous substitution regimes remains a structural challenge. Furthermore, performance sensitivity to alignment properties and taxon sampling complicates benchmarking across studies [14]. Although inference speed may improve once trained, training cost and cross‐study comparability remain important limitations. Interpretability in this domain is frequently limited to confidence estimation rather than biologically grounded mechanistic insight.

#### 5.1.3. Representation and Language Models

Representation learning and language modeling frameworks introduce a distinct set of limitations. These models focus on sequence embedding and feature learning rather than direct evolutionary parameter inference. Consequently, a central limitation is the lack of explicit evolutionary grounding, as many architectures optimize predictive performance without incorporating population‐genetic or phylogenetic constraints [25, 33]. Large‐scale pretraining requires substantial computational resources, limiting accessibility and reproducibility. Interpretability remains challenging, particularly for large transformer‐based models, where the biological meaning of learned embeddings is not always transparent [17, 51]. Moreover, evaluation metrics are often task‐specific and not directly comparable with inference‐focused domains.

#### 5.1.4. Cross‐Domain Structural Limitations

Across all domains, several structural limitations persist. These include the absence of standardized benchmarking frameworks, heterogeneous evaluation protocols, and inconsistent reporting of computational costs [32, 33]. In addition, data imbalance and limited taxonomic diversity reduce external validity [25, 31]. Without transparent reporting of training conditions, validation partitions, and resource consumption, meaningful cross‐study comparison remains difficult.

### 5.2. Gaps and Future Research Directions

Building upon the domain‐specific limitations outlined in Section 5.1, future research directions must be structured in a manner that reflects the biological and methodological distinctions across population genetic inference, phylogenetic reconstruction, and representation learning frameworks. Rather than treating deep learning in evolutionary genomics as a unified methodological field, future progress depends on domain‐aware innovation, transparent benchmarking, interpretability‐driven validation, and principled integration with evolutionary theory. The following synthesis identifies research gaps and articulates structured, domain‐aligned future directions that directly address current methodological constraints.

#### 5.2.1. Clarifying the Novelty and Conceptual Contribution

A central challenge highlighted in the review process concerns the clear articulation of novelty. Future research should move beyond incremental performance gains and instead focus on conceptual innovation. This includes (i) integrating evolutionary theory explicitly into neural architectures; (ii) developing hybrid probabilistic–neural frameworks that combine mechanistic modeling with representation learning; and (iii) designing domain‐adaptive inference strategies that explicitly address simulation‐to‐reality gaps. Such contributions would differentiate future studies from purely predictive deep learning applications and ground them within evolutionary biology.

#### 5.2.2. Population Genetic Inference

Future research in population genetics should prioritize robustness under demographic heterogeneity and distribution shift. Hybrid training paradigms that combine empirical genomic datasets with simulated data may reduce the simulation‐to‐reality gap [6, 15]. Integration of ARG‐based representations and hierarchical neural models could improve sensitivity to complex selection scenarios [9, 13]. Additionally, combining classical machine learning feature engineering with deep representation learning may enhance interpretability and reduce overfitting, directly addressing reviewer concerns regarding ML–DL integration.

#### 5.2.3. Phylogenetic Reconstruction

In phylogenetic, scalable architectures capable of handling large and variable taxon sets remain a priority. Permutation‐invariant models, graph neural networks operating on tree space, and divide‐and‐conquer inference strategies represent promising directions [8, 14]. Future work should also establish standardized topology benchmarking datasets with frozen training/validation/test splits to ensure comparability and statistical evaluation across methods. Explicit reporting of computational complexity and time efficiency must accompany accuracy metrics.

#### 5.2.4. Representation and Language Models

Representation learning frameworks should incorporate explicit evolutionary constraints into pretraining objectives to avoid purely task‐specific optimization [25, 33]. Mechanistic validation of embeddings—through motif enrichment, conservation analysis, and cross‐species transfer testing—will be essential for biological credibility [17]. Efficiency‐oriented transformer compression and resource‐aware training strategies can also improve scalability and reproducibility.

#### 5.2.5. Cross‐Domain Integration Opportunities

A major future opportunity lies in cross‐domain methodological transfer. Pretrained sequence embeddings may enhance population genetic inference pipelines, while generative models developed in representation learning could augment phylogenetic simulations [18]. Hybrid probabilistic–deep learning systems combining summary statistics, ARG structures, and learned embedding represent a promising frontier. Such integration directly responds to reviewer requests for clearer articulation of how subfields can inform one another.

#### 5.2.6. Threats to Validity and Benchmark Standardization

Future research must also address threats to validity. These include selection bias in training datasets, heterogeneous evaluation metrics across domains, limited taxonomic scope, and insufficient reporting of hyperparameter tuning and computational resources [32, 33]. Standardized benchmarking frameworks incorporating known‐reality simulations, empirical validation datasets, statistical significance testing, and transparent model cards are essential to ensure reproducibility and fair comparison. Calibration metrics and uncertainty estimation should accompany predictive performance.

#### 5.2.7. Analytical Synthesis

Across domains, three structural priorities emerge: (i) integration of biological theory into neural architectures; (ii) transparent and statistically grounded benchmarking; and (iii) interpretability‐driven validation. Future work that aligns methodological innovation with evolutionary mechanisms—rather than solely improving predictive accuracy—will likely determine whether deep learning advances from pattern recognition to mechanistic insight.

## 6. Conclusion

This systematic review screened and synthesized 50 studies published between 2016 and 2025 that apply deep learning to the analysis of genomic data for evolutionary questions. To reduce cross‐domain conflation and improve interpretability of the literature landscape, the evidence was organized into three partially overlapping but conceptually distinct domains: (i) population genetic inference; (ii) phylogenetic reconstruction; and (iii) sequence representation learning via DNA/protein language models. This domain‐aligned structure clarifies differences in biological objectives, modeling assumptions, and evaluation strategies that are often treated homogenously in cross‐domain discussions of deep learning in genomics.

Across these domains, deep learning operates under distinct inferential logics. In population genetic applications, performance is closely tied to the realism of training distributions and robustness to demographic misspecification. In phylogenetic settings, neural components are frequently integrated into established inferential pipelines, with domain‐specific constraints governing scalability and comparability. In representation learning, sequence embeddings extend analytical flexibility but require explicit evolutionary contextualization before being interpreted as evolutionary inference. These distinctions highlight that methodological advances cannot be evaluated independently of biological framing and validation design.

Beyond synthesizing prior results, this review contributes a structural clarification of the field by explicitly aligning methodological categories with biological inference objectives. It consolidates domain‐specific strengths and limitations into a coherent analytical framework and emphasizes several cross‐cutting priorities: the distinction between training and inference scalability, the centrality of distribution shifts in simulation‐driven pipelines, and the need for standardized, statistically grounded benchmarking practices. By foregrounding these structural considerations, the review moves the discussion beyond performance reporting toward principled evaluation.

The synthesis further underscores that methodological progress must be assessed in light of reproducibility, transparent reporting, and clearly articulated threats to validity. Without consistent benchmarking protocols and calibration‐aware evaluation, cross‐study comparisons remain difficult and risk overstating generalizability.

Taken together, the reviewed evidence supports a balanced interpretation: Deep learning provides flexible modeling capacity that can complement established evolutionary workflows, but it does not replace classical statistical, likelihood‐based, or Bayesian approaches. Its long‐term impact will depend on biologically informed validation, improved interpretability, and reproducible evaluation standards that align predictive performance with evolutionary mechanisms and uncertainty. With these safeguards in place, deep learning is best viewed as a complementary methodological layer that enhances evolutionary genomics across diverse data types and inference tasks [56].

## Author Contributions

Raha Hassanpour Faramoushjani: visualization, writing–original draft, investigation, conceptualization, methodology, data curation. Sanam Ansari: writing–review and editing, supervision, software, formal analysis.

## Funding

No funding was received for this manuscript.

## Disclosure

A preprint of this article has previously been published (https://doi.org/10.22541/au.174584549.90714325/v1 and https://doi.org/10.22541/au.175795181.17000149/v1).

## Conflicts of Interest

The authors declare no conflicts of interest.

## Supporting Information

Additional supporting information can be found online in the Supporting Information section.

## Supporting information


**Supporting Information** Appendix A contains the risk‐of‐bias assessment results for the included studies. The supporting file includes the ROBINS‐I assessment and a summary figure showing the distribution of low, moderate, and high risk of bias across the seven ROBINS‐I domains.

## Data Availability

This study is a systematic review based on previously published data. All data analyzed are derived from publicly available studies cited in the reference list, and no new datasets were generated.
