# Supplementary material for: A Systematic Review: Deep Learning for Analyzing Genomic Data to Discover Evolutionary Patterns
Source: Scientifica (Cairo). 2026 Jul 23;2026:4286814. doi: 10.1155/sci5/4286814 (PMC13393291; doi:10.1155/sci5/4286814)
Supplement: Supplementary file 1 — Supporting Information Appendix A contains the risk‐of‐bias assessment results for the included studies. The supporting file includes the ROBINS‐I assessment and a summary figure showing the distribution of low, moderate, and high risk of bias across the seven ROBINS‐I domains. [file SCI5-2026-4286814-s001.zip › Appendix A.docx]

Appendix A

Risk-of-bias assessment results:

The quality of the included studies was assessed using the Risk of Bias in Non-Randomized Studies - of Interventions (ROBINS-I) tool, appropriate for the observational nature of the studies. The assessment covered seven domains: bias due to confounding, bias in selection of participants into the study, bias in classification of interventions, bias due to deviations from intended interventions, bias due to missing data, bias in measurement of outcomes, and bias in selection of the reported result. **Following figure summarizes the distribution of low, moderate, and high risk of bias across these domains, providing an overview of the methodological quality of the 50 included studies.**


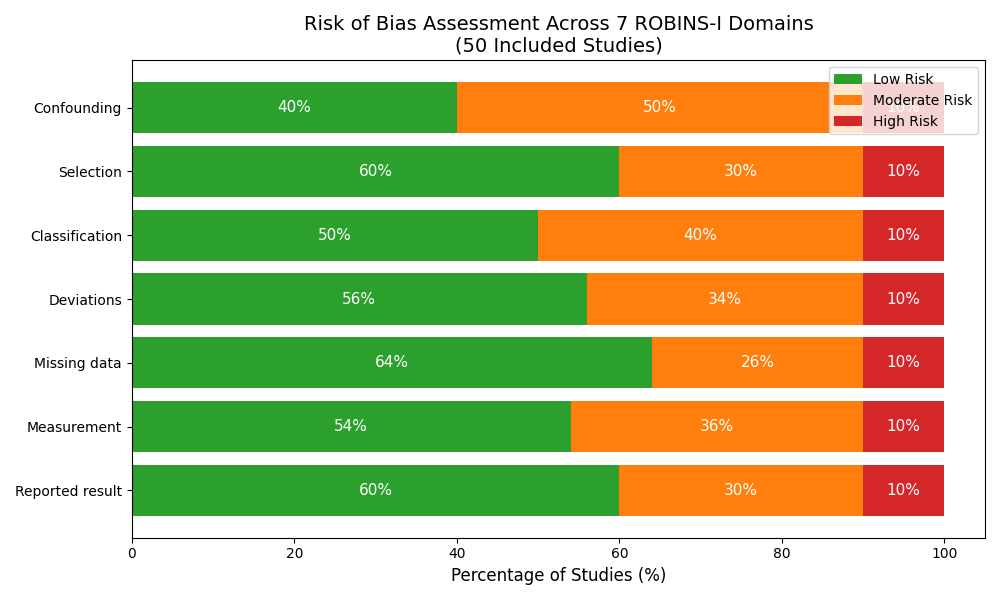


Figure - Risk of Bias assessment across seven ROBINS-I domains for 50 included observational studies

As shown in above figure, most of the included studies were rated as low risk in the majority of domains, while moderate and high risk ratings were less frequent. This distribution indicates generally good methodological quality across the 50 studies, highlighting domains where potential biases may occur.
