# Supplementary figures and images for: A Systematic Review: Deep Learning for Analyzing Genomic Data to Discover Evolutionary Patterns
Source: Scientifica (Cairo). 2026 Jul 23;2026:4286814. doi: 10.1155/sci5/4286814 (PMC13393291; doi:10.1155/sci5/4286814)

# Risk of Bias Assessment Across 7 ROBINS-I Domains (50 Included Studies)

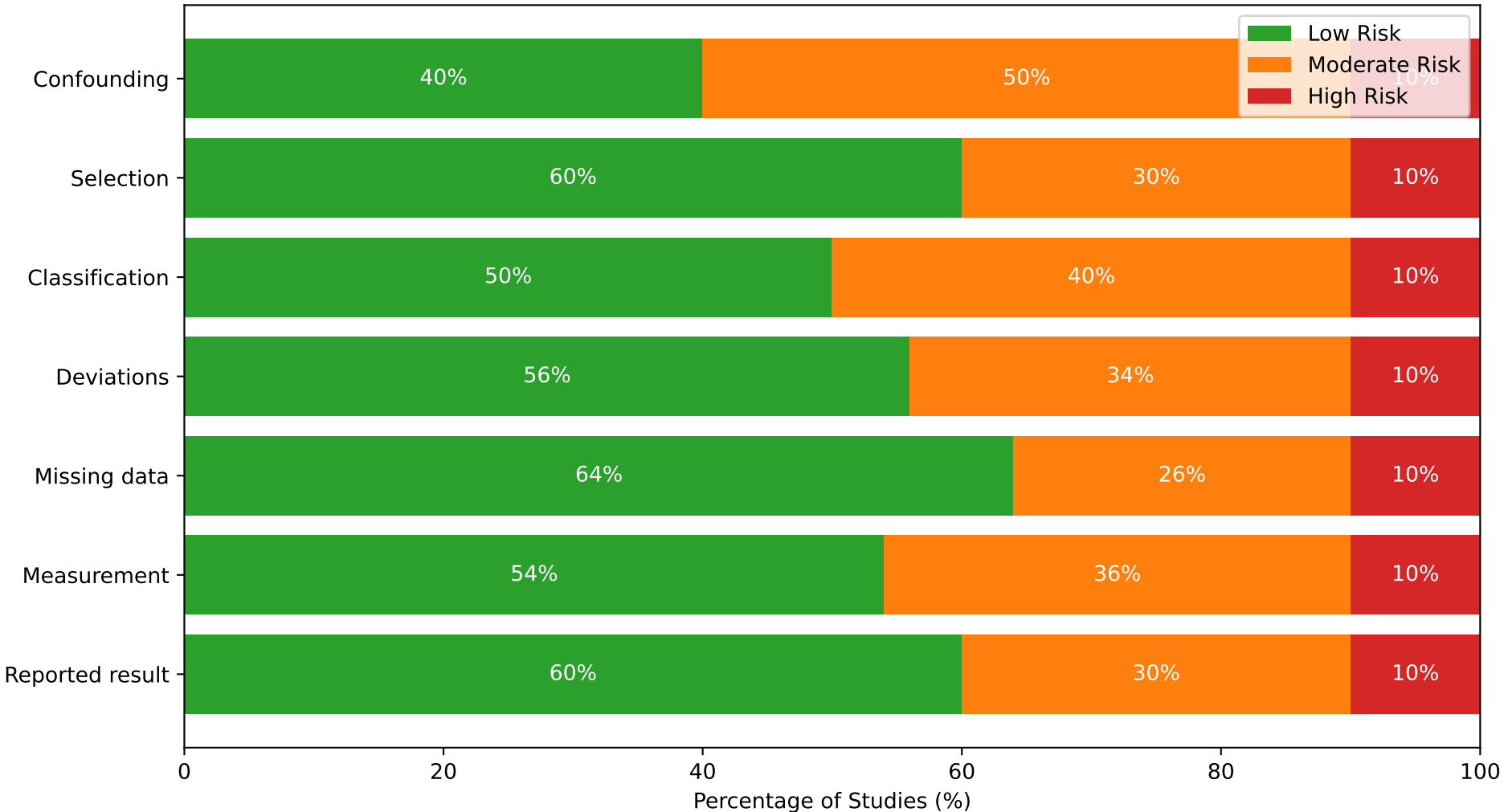

Supplement: Supplementary file 1 — Supporting Information Appendix A contains the risk‐of‐bias assessment results for the included studies. The supporting file includes the ROBINS‐I assessment and a summary figure showing the distribution of low, moderate, and high risk of bias across the seven ROBINS‐I domains. [file SCI5-2026-4286814-s001.zip › appendix.pdf]
